# Trends in the use of antiasthmatic medications in Morocco (1999–2010)

**DOI:** 10.1186/2193-1801-2-82

**Published:** 2013-03-05

**Authors:** Imane Ghanname, Samir Ahid, Ghizlane Berrada, Abdelmjid Belaiche, Mohamed Hassar, Yahya Cherrah

**Affiliations:** 1 Department of Pharmacology and Toxicology, Faculty of Medicine and Pharmacy, Research team of Pharmacoepidemiology & Pharmacoeconomics, Rabat, Morocco; 2 PHARMED, Casablanca, Morocco

**Keywords:** Antiasthmatic, Average monthly expenditure, Consumption, Defined Daily Dose (DDD), Morocco

## Abstract

**Background:**

Asthma is a big public health problem in Morocco. The drug therapy existing in Morocco is currently insufficient because of the low purchasing power and the low health insurance coverage available to the average citizen in Morocco. In this study we evaluated the consumption of antiasthmatics in Morocco during the period 1999–2010, the classes of used drugs and the generics’ market share.

**Methods:**

We used sales data from the Moroccan subsidiaries of the IMS Health “Intercontinental Marketing Service”. The consumption volume was converted to Defined Daily Doses (DDDs).

**Results:**

During 1999–2010, antiasthmatics’s consumption increased from 3.91 to 14.47 DDD per 1000 inhabitants per day. In 2010, the association Beta-2-mimetic-Glucocorticosteroids were the most consumed (8.53 DDD/1000 Inhabitants/day) followed by the short-acting inhaled Beta-2-mimetic (4 DDD/1000 Inhabitants/day) and inhaled Glucocorticosteroids alone accounted for 1.13 DDD/1000 Inhabitants/day. The largest consumption share in volume was held by the short-acting inhaled Beta-2-mimetic (42%) followed by the combination Beta-2-mimetic-Glucocorticosteroids (38%). Between 1999 and 2010, the market for generic antiasthmatics increased from 1.84 to 2.18 DDD/1000 Inhabitants/day. The ratio of the monthly average cost of treatment to the minimum wage in Morocco decreased from 10.8% in 1999 to 7.11% in 2010.

**Conclusion:**

Antiasthmatics’ consumption in Morocco has undergone significant changes between 1999 and 2010. However, the availability of these drugs expressed as the Average Monthly Expenditure/Guaranteed Minimum Wage ratio improved. Despite this, the use of antiasmathics in Morocco remains low.

## Background

Asthma is a global public health problem. It affects about 300 million people (Global Strategy for Asthma Management and Prevention 2011, Bateman et al. 2008). Its prevalence varies from 1% to 18% of the population (Masoli et al. 2004, Urrutia et al. 2007, Von Mutius 2007). It is increasing in some countries such as Taiwan (Yan et al. 2005), while remaining stable in other countries, such as Spain (Carvajal-Urueña et al. 2005, García-Marcos et al. 2004, Asher et al. 2006). On the other hand, Germany has seen a decrease of 2% per year (Masoli et al. 2004). The World Health Organization (WHO) estimates that 255,000 people died of asthma in 2005 (World Health Organization Collaborating Center for Drug Statistics Methodology 2011).

New approaches in the management of asthma stress the importance of controlling the disease. Indeed, the consequences in terms of quality of life and cost (“Burden of Disease”) of asthma are important and it is necessary to take them into account (Global Strategy for Asthma Management and Prevention 2011, Masoli et al. 2004, Beasley 2002).

The number of studies has increased substantially in developed countries and has led to a better understanding of the overall cost of the disease (Barnes et al. 1996, Weiss et al. 1992). Exacerbations of the disease and resulting hospitalizations, in addition to their impact on the evolution of drug consumption, impose a significant financial burden on the patients (Godard et al. 2002). The higher costs result from an increased use of the new antiasthmatic associations as they are more efficient (Shrewsbury et al. 2000). Their costs are not negligible and have been the subject of many studies in several industrialized countries (Beasley 2002, Weiss et al. 1992). In the USA, the overall cost of medical treatment for asthma patients is estimated to be US$ 5.8 billion, including US$ 5.1 billion of direct costs (Smith et al. 1997). In France, the overall cost of asthma is estimated at 1.5 billion Euros (Com-Ruelle et al. 2002), while in Africa, we don’t have no pharmaco-economic study of asthma.

These reasons prompted us to conduct a study about the use of antiasthmatic medications in Morocco during the period 1999–2010 to have an idea about the consumed antiasthmatics and the cost for each one. The place of generic drugs in anti-asthma treatment was also studied.

## Materials and methods

We used sales data from the Moroccan subsidiary of IMS Health. The data for drugs sold by private pharmacies were utilized since it represents 90% of the global pharmaceutical consumption in Morocco. The study involved the main antiasthmatic classes with the INNs (International Non-proprietary Name) shown in Table 1.

**Table 1:** **Evolution of consumption in DID of the different families of antiasthmatic drugs**

	***1999***		***2006***		***2010***	
	***DID***	***%***	***DID***	***%***	***DID***	***%***
**Short-acting inhaled Beta-2-mimetic**	1.95	48.87	3.46	51.18	4	27.64
**Long-acting inhaled Beta-2-mimetic**	0.03	0.76	0.07	1.03	0.04	0.48
**Systemic Beta-2-mimetic**	0.27	6.9	0.33	0.33	0.35	2.42
**Inhaled Glucocorticosteroids alone**	0.71	18.15	1.11	16.42	1.13	7.81
**Association Beta-2-mimetic /Glucocorticosteroids**	–––	––––	1.32	19.53	8.53	58.95
**Injectable Xanthines**	0.95	24.30	0.47	6.95	0.39	2.70
	***Σ = 3.91***	***100%***	***Σ = 6.76***	***100%***	***Σ = 14.47***	***100%***

DID: **D**DD/1000 **I**nhabitants /**D**ay.

Data from IMS sales in units were converted to DDDs/1000 Inhabitants per day (DDD/1000 Inhabitants/day = [Number of DDD/Number of days * Number of inhabitants]*1000), using the proposed definition of DDDs by WHO (WHO Collaborating Centre for Drug Statistics Methodology 2009), which is “an estimate of the maintenance’s average dose per day for a drug used for its main indication for an average adult”. This unit of measurement allows comparisons at an international level by eliminating the difficulties associated with the heterogeneity of dosage forms, presentations and dosages of drugs across countries.

A DDD is assigned to each dosage form of a drug. Thus, for each drug of the Anatomical Therapeutic Chemical system (ATC), a daily dose is given the value of a DDD. In this study we used the ATC system version of European Pharmaceutical Market Research association “EphMRA” (WHO Collaborating Centre for Drug Statistics Methodology 2009).

Drug usage (DDDs) = Items issued *Amount of drug of item / WHO DDD Measure (Sommet et al. 2005).

For the active ingredients not included in the WHO ATC classification, as well as associations, the recommended dose of the Vidal® dictionary was used as DDD.

We calculated the evolution of Moroccan Medium Prices (“MMP”) of antiasthmatics in Moroccan Dirham (MAD) to show the impact of generics and the availability of new molecules on this mean price (1 Euro = 11 MAD).

For the socio-economic aspect of the study, we calculated the Average Monthly Expenditure (“AME”) (AME = [(Prescripted daily doses * Price of presentation)/ Number of units per presentation] * 30.42) of different antiasthmatics compared to the Guaranteed Minimum Wage (“GMW”) (High Commission of Planning-HCP 2010) in Morocco in order to get the share of the cost of drugs in the household spending.

Data were entered and analyzed using SPSS 13.0. Quantitative variables were expressed as arithmetic and weighted averages and as standard deviation. These averages were compared by using the ANOVA test. The Pearson correlation coefficient was used for quantitative variables. The significance level was set at p < 0.05.

## Results

**Figure 1:**
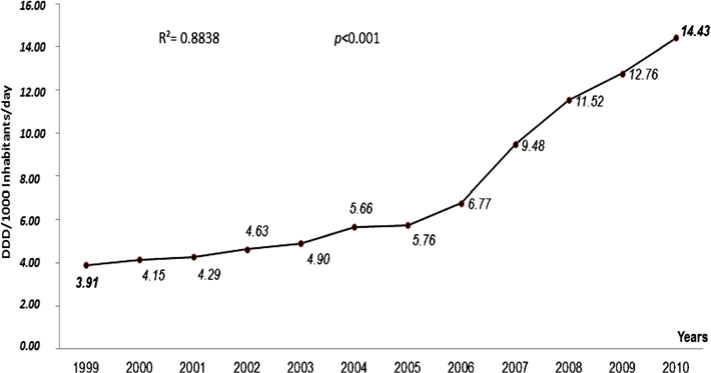
**Evolution of consumption in DDD/1000 Inhabitants/day of all antiasmathic drugs in Morocco.**

During the study period of 1999 to 2010, the total consumption of antiasthmatics went from 3.91 to 14.47 DDD/1000 Inhabitants/day (DID), a 370% increase (Figure 1).

In 2010 and per family, Beta-2-mimetic associated with Glucocorticosteroids were the most consumed 8.53 DID for each one, followed by the short-acting inhaled Beta-2-mimetic (4.0 DID) and Glucocorticosteroids (1.13 DID). The consumption of the other classes of drugs, namely the Beta-2-mimetic for systemic use and the long-term inhaled Beta-2-mimetic, witnesses a much slower evolution. However, there was a marked decline in the consumption of the injectable Xanthines from 0.95 DID in 1999 to 0.39 DID in 2010 (−58.95%) (Table 1).

In 2010, the most consumed products were the association Salmeterol + Fluticasone (6.05 DID), followed by the short-acting inhaled Salbutamol (3.94 DID) and the association Budesonide + Formeterol (2.47 DID). We must note that during the period of our study, new drugs have been introduced into the market and became the most widely consumed in Morocco for 2010 (Table 2).

**Table 2:** **Consumption trends of antiasthmatic drugs in INN**

***Classes/INN***	***ATC***	***DDD/1000H/D***		
		***1999***	***2006***	***2010***
**Short-acting inhaled Beta-2-mimetic**				
**Salbutamol**	R03AC02	**1.70**	**3.42**	3.94
**Terbutaline**	R03AC03	0.12	0.04	0.06
**Fenoterol**	R03AC04	0.06	-	-
**Pirbuterol**	R03AC08	0	-	-
**Orciprenaline**	R03AB03	0.06	-	-
**Systemic Beta-2-mimetic**				
**Salbutamol**	R03CC02	0.23	0.31	0.33
**Terbutaline**	R03CC03	0.04	0.03	0.02
**Long-acting inhaled Beta-2-mimetic**				
**Formeterol**	R03AC12	0.01	0.07	0.04
**Salmeterol**	R03AC13	0.02	-	-
**Association Beta-2-mimetic /Glucocorticosteroids**				
**Fluticasone + Salmeterol**	R03AK06	-	1.08	**6.05**
**Budesonide + Formeterol**	R03AK07	-	0.24	2.47
**Inhaled Glucocorticosteroids alone**				
**Bedomethasone**	R03BA01	0.65	0.95	1.01
**Budesonide**	R03BA02	0.03	0.01	
**Fluticasone**	R03BA05	0.03	0.15	0.12
**Injectable Xanthines**				
**Theophylline**	R03DA04	0.89	0.43	0.34
**Diprophylline + Tiemonium iodure**	R03DA51	0.05	0.05	0,.05

INN: **I**nternational **N**on-proprietary **N**ame / ATC : **A**natomical, **T**herapeutic and **C**hemical classification / DID : **D**DD/1000 **I**nhabitants /**D**ay.

Similarly, the number of antiasthmatic commercial specialties in Morocco rose from 27 in 1999 to 36 in 2010, and the number of pharmaceutical presentations of antiasthmatics also rose from 57 in 1999 to 68 in 2010 (an increase of 19.30%) (Table 3).

**Table 3:** **Evolution of the monthly average cost of antiasthmatic drugs compared to guaranteed minimum wage moroccan asthma**

	***1999***				***2006***				***2010***			
	***AME***	***AME/GMW***	***NS***	***NP***	***AME***	***AME/GMW***	***NS***	***NP***	***AME***	***AME/GMW***	***NS***	***NP***
**Short-acting inhaled Beta-2-mimetic**	66.5	4.01	7	9	77.5	3.86	9	11	38.61	1.83	8	12
**Long-acting inhaled Beta-2-mimetic**	425.5	25.64	2	3	265.04	13.19	3	4	209.17	9.92	2	2
**Systemic Beta-2-mimetic**	76.4	4.60	4	14	68.61	3.41	7	18	71.27	3.38	6	13
**Inhaled Glucocorticosteroids alone**	192.9	11.82	5	9	147.06	7.32	8	15	144.66	6.86	11	15
**Association Beta-2-mimetic /Glucocorticosteroids**	-	-	-	-	363.02	18.07	2	6	209.20	9.92	4	13
**Injectable Xanthines**	46.1	2.77	9	22	54.73	2.72	9	18	56.07	2.67	5	13
	***(161.40 ± 158.32)***	***(9.77 ± 9.55)***	27	57	***(162.66 ± 125.39)***	***(8.10 ± 6,24)***	38	72	***(121.5 ± 76.93)***	***(5.76 ± 3.65)***	36	68

AME : Average Monthly Expenditure (MAD).

GMW : Guaranteed Minimum Wage (MAD) (High Commission of Planning-HCP 2010).

AME/GMW : In percentage.

NS : Number of Specialties / NP : Number of Pharmaceutical Presentations.

1999 : GMW = 150.89 € AME/GMW = 9.72% CI = [2.12% ; 21.56%] with 95%.

2006 : GMW = 182.66 € AME/GMW = 8.10% CI = [1.54% ; 14.65%] with 95%.

2010 : GMW = 191.74 € AME/GMW = 5.76% CI = [1.94% ; 9.59%] with 95%.

**Figure 2:**
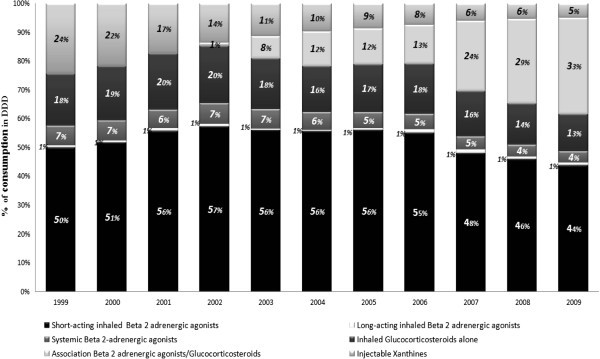
**Consumption trends segmentation of antiasthmatic drugs in DDD.**

In 1999, antiasthmatics consumption, in DDD, was represented by the short-acting inhaled Beta-2-mimetic (50%), systemic Xanthines (24%), Glucocorticosteroids (18%), Beta-2-mimetic for systemic use (7%) and the long-acting inhaled Beta-2-mimetic (1%). But in 2010, it was 42% for the short-acting inhaled Beta-2-mimetic, 38% for the combination Beta-2-mimetic-Glucocorticosteroids, 12% for the Glucocorticosteroids, 4% for the systemic Xanthines and 4% for the Beta-2-mimetic for systemic use (Figure 2).

From 1999 to 2001, the antiasthmatics’ generic market increased from 1.84 to 2.18 DID. In 2010, inhaled Glucocorticosteroids ranked first for generic consumption (0.83 DID), followed by the short-acting inhaled Beta-2-mimetic (0.73 DID). The drugs resulting from the association Beta-2-mimetic-Glucocorticosteroids accounted for the largest share of antiasthmatics consumption (3.40 DID).

The AME of all antiasthmatics decreased gradually from 161.48 MAD in 1999 to 121.50 MAD in 2010, a 24.76% decrease.

The ratio AME/GMW decreased during the study period, falling from 9.77% in 1999 to 5.76% in 2010. However, this though decrease was not statistically significant for all antiasthmatic classes (p = 0.79) (Table 3).

## Discussion

This is the first study in Morocco that analyzes antiasthmatic drug consumption data. These drugs are mainly prescribed for certain respiratory diseases especially asthma and chronic obstructive pulmonary disease (COPD).

From 1999 to 2010, consumption of antiasthmatic drugs sold in the pharmacies has significantly increased by an average of 13.07% per year. This evolution in consumption over a period of 11 years is marked by a two-fold increase in the use of inhaled Beta-2-mimetic specifically in the use of associations as they have been shown very effective (Shrewsbury et al. 2000). Consumption of other classes, namely inhaled Glucocorticosteroids and other systemic antiasthmatics remained stable over this period.

Viens et al. showed that in 2004 the antiasthmatic consumption in Germany and France were respectively 60 and 62 DID for a prevalence of asthma at about 7%. In the UK, where the prevalence is between 15.3% and 18.4%, the consumption is 130 DID. Surprisingly, Spanish and Italian consumption levels of 81 DID and 65 DID may seem high for a low prevalence of asthma, 5.7% and 4.5% respectively. In 2004, Moroccan consumption was estimated at 5.66 DID. The prevalence is not known exactly because of a lack of epidemiological studies.

In Morocco, the evolution of consumption of Beta-2-mimetic class, in both units sold and dosage units, is due to the high consumption of short-acting Beta-2-mimetic, in particular Salbutamol (Aåt-Khaled and Enarson 2005).

Inhaled Glucocorticosteroids are the first line treatment of symptomatic asthma (Pujet and Evano-Celli 1995, High Authority for Health (HAS) 2004, Swiss Group for Pediatric Pneumology work SAPP 2004). The original and the generic Beclomethasone represent the major share of the consumption of this class. In 2010, the Moroccan market in DDD is represented by Beclomethasone (89.38%), Fluticasone (10.61%) and by Budesonide (less than 1%). Furthermore, the increased consumption of inhaled Glucocorticosteroids seems to be in line with the various recommendations advocating Glucocorticosteroids therapy as soon as possible (Coignard et al. 2001).

In 2004, in France and Italy, the use of fixed combination of inhaled Beta-2-mimetic and Glucocorticosteroids is the most common treatment (respectively 38% and 37% of the consumption of antiasthmatics) (Viens et al. 2007). In Morocco, these products, marketed in 2002, showed an extremely rapid growth in sales as they are easier to use by patients in need, but they were only prescribed as a second line of treatment (Cuerq et al. 2008). In parallel, we found a decrease in consumption of the Xanthines, including Theophylline, which is the most consumed product of this class. This change is normal because of the small margin of tolerance and the individual variability of the metabolism of Theophylline (Coignard et al. 2001).

A study of fixed-dose combinations usage conducted by the French Transparency Committee in 2006 found that half of the asthma patients treated with fixed combinations had no previous treatment with inhaled Glucocorticosteroids alone (HAS 2006). Thus, some patients do not receive basic treatment with inhaled Glucocorticosteroids, while a great number are prescribed as first treatment associations of molecules that are considered second line therapy.

In Morocco, the reduction in consumption of the short-acting Beta-2-mimetic alone is not accompanied by an increase in the consumption of Glucocorticosteroids and the long acting Beta-2-mimetic that one would have expected, since these increases would reflect a better overall therapeutic management.

In 2006, the antiasthmatic market in Morocco was dominated by the consumption of the Beta-2-mimetic (61%), followed by the Glucocorticosteroids (18%). This is in line with the findings in European countries. According to the study of ESSEC, this convergence could be explained by the standardization of medical practices. European recommendations are generally adopted by Moroccan practitioners (Viens et al. 2007).

In 2002, according to Godard et al., direct annual costs in France ranged from € 263 for the stage I to € 2782 for the stage IV. The costs of hospitalizations were excluded because of the difficulty to assess them at that time. Drugs accounted for 58% to 75% of the direct annual costs depending on the stage (Vergnenègre et al. 2008). In Morocco, the monthly cost of anti-asthma treatment between 1999 and 2010 decreased from 161.48 ± 158.32 MAD (or 14.68 ± 14.39 €) to 121.50 ± 76.3 MAD (or 11.05 ± 6.40 €). The largest share of consumption was represented by inhaled Glucocorticosteroids.

The economic analysis of published clinical trials using inhaled Glucocorticosteroids shows an increase in the cost of drugs and, in some cases, the total costs of asthma management, but their use results in better control of exacerbations and of other symptoms (Shrewsbury et al. 2000, Pauwels et al. 1997, O’Byrne et al. 2001, Greening et al. 1994).

In Morocco, the monthly average cost decreased by 24.72% between 1999 and 2010. This is explained by the use of the fixed-dose combination of inhaled Beta-2-mimetic and Glucocorticosteroids and the share of generic drugs which represents about 37.9% of the antiasthmatic market. The short-acting inhaled Beta-2-mimetic were the cheapest class of the drugs used in monotherapy (Ind et al. 2003), decreasing from a monthly average of 66.5 MAD in 1999 to 38.61 MAD in 2010.

During our study period we noted a decrease in the ratio AME/GMW. This is explained by a decrease in AME, but mainly by an increase of 4.73% of the GMW (Secrétariat général du gouvernement 2004). This ratio was estimated at 9.77% in 1999 and decreased to 5.76% in 2010. Health spending has more than doubled, from 1.09% to 2.50% of GDP. This is likely to have an impact on access to care (High Commission of Planning-HCP 2010).

The minimum annual cost of drugs to treat a moderate asthma case with the same standard treatment recommended by the International Union against Tuberculosis and Lung Disease varied in 2002 from one country to another: US$ 54 (€ 37.95) in Algeria, US$ 288 (€ 202.4) in Sudan and US$ 650 (€ 456.82) in Kuwait (Aåt-Khaled and Enarson 2005). The high price of asthma drugs is a major barrier to adhere to long-term treatments, especially for disadvantaged groups of patients. The majority of these patients have insufficient income to purchase their medications on a regular basis and very few have health care insurance coverage (Secrétariat général du gouvernement 2008, Aåt-Khaled et al. 2001).

In recent years, great efforts have been deployed in Morocco by the Ministry of Health to develop a national policy of encouraging the manufacture of generic drugs in order to facilitate access to treatment. Despite this, the use of antiasthmatic generic drugs remains limited as the original products, still under patent, still dominate the market.

This study, showing the consumption profile of antiasthmatic drugs in Morocco during the decade 1999–2010, has some limitations. The DDD unit used to calculate the consumption is an approximate unit of measurement and it does not necessarily reflect the daily dose consumed. Also, it does not take into account the severity of asthma stages. We have to keep in mind that asthma is indicated for the treatment of asthma, which affects all age groups with a female predominance, as well as other diseases, such as chronic pulmonary obstructive, which is most often diagnosed in adult men and smokers, or chronic bronchitis (Coignard et al. 2001). Thus, we can not infer from the study data that all drugs have been used exclusively to treat asthma. In addition, the data don’t contain no information on adherence. Therefore, the term “consumption” is used figuratively and, at no time, we can assume that the purchased drug was effectively consumed.

These limitations show that this method may be useful as a first line indicator to monitor the use of a drug or a class of drugs. Other methods may be necessary to monitor more precisely the real situation, factoring in the characteristics of the population studied. Finally, we have to use the ATC/DDD method through choosing a relevant observation period (e.g.: a period long enough to control the seasonality bias that may influence the findings) (Abou-Atmé et al. 2006).

## Conclusion

In Morocco, antiasthmatics consumption has increased between 1999 and 2010. Our findings also highlight the need for other studies (epidemiological, prescribing patterns, management protocols, access to care) in order to fully explain the differences in consumption of antiasthmatics in Morocco.
